# Vancomycin Re-Exposure-Induced Immune Hemolytic Anemia

**DOI:** 10.7759/cureus.106679

**Published:** 2026-04-08

**Authors:** Yiwen Jiang, Yanfeng Gan, Ziyu Li, Yan Zhong, Xiaosi Li

**Affiliations:** 1 Department of Pharmacy, Hospital of Chengdu Office of People's Government of Xizang Autonomous Region, Chengdu, CHN; 2 Department of Orthopedics, Hospital of Chengdu Office of People's Government of Xizang Autonomous Region, Chengdu, CHN

**Keywords:** adverse drug reaction, direct antiglobulin test, immune hemolytic anemia, periprosthetic infection, vancomycin

## Abstract

Vancomycin-induced immune hemolytic anemia is a rare but potentially life-threatening adverse drug reaction that is often underrecognized. We report a case of a 76-year-old male who developed an infection following hip arthroplasty and received vancomycin for the first time in September 2025, resulting in a decrease in hemoglobin from 14.1 g/dL to 10.4 g/dL with normal platelet counts. After readministration in March 2026, hemoglobin decreased by 8.2 g/dL, reticulocyte count rose to 7.00%, and the direct antiglobulin test was positive, confirming drug-induced immune hemolytic anemia (DIIHA). Hemoglobin gradually recovered after discontinuation, but reactive thrombocytosis and coagulation abnormalities occurred. This case highlights that vancomycin can induce immune hemolytic anemia, with more severe and rapid progression upon re-exposure.

## Introduction

Vancomycin is a first-line agent for the treatment of Gram-positive bacterial infections (particularly methicillin-resistant Staphylococcus aureus and methicillin-sensitive Staphylococcus aureus) and is widely used in orthopedic infections [1]. Common adverse reactions of vancomycin include infusion reaction, nephrotoxicity, and ototoxicity [2]. The infusion reaction is a non-immune-mediated histamine release, manifesting as erythema on the face, neck, and upper trunk, and is correlated with infusion rate [3]. In addition, vancomycin can also induce a drug reaction with eosinophilia and systemic symptoms (DRESS) syndrome, a severe hypersensitivity reaction characterized by rash, fever, eosinophilia, and involvement of multiple organs [4]. Vancomycin-induced immune hemolytic anemia is a rare but potentially life-threatening complication [5].

The incidence of drug-induced immune hemolytic anemia (DIIHA) is approximately 2.44 per million annually. However, due to the underreporting of severe cases, the actual incidence may be underestimated [6]. The pathogenesis of DIIHA primarily involves drug-dependent antibodies (typically IgG) binding to erythrocyte membranes, thereby activating the complement system and leading to immune-mediated destruction of erythrocytes [7]. Over 140 drugs have been reported to potentially induce immune-mediated hemolytic anemia. Historically, methyldopa and high-dose penicillin were the most common culprits, while currently, ceftriaxone, other cephalosporins, piperacillin, and non-steroidal anti-inflammatory drugs (NSAIDs) are the predominant agents [8].

This report presents a case of a 76-year-old male patient with periprosthetic infection who developed characteristic hemolytic manifestations after re-exposure to vancomycin after six months. This report, along with a literature review, discusses the clinical features, diagnostic key points, and treatment strategies to enhance clinical awareness and vigilance regarding vancomycin-induced immune hemolytic anemia.

## Case presentation

A 76-year-old male presented with no specific complaints related to hemolysis at the time of hospital admission. He denied dark-colored urine, pallor, jaundice, fever, tachycardia, or abdominal pain. His past medical history included grade 2 hypertension for six years (maximum systolic blood pressure 145 mmHg, diastolic not documented) treated with felodipine 5 mg once daily, paroxysmal atrial fibrillation, and left inguinal hernia. He had no history of allergies, hepatitis, tuberculosis, or other infectious diseases. Surgical history included right total hip arthroplasty for femoral head necrosis in November 2022, right hip joint infection debridement in June 2025, another debridement with catheter irrigation in July 2025, and subsequent catheter removal. In August 2025, the patient had received rifampicin (600 mg once daily) and moxifloxacin (400 mg once daily) for a Propionibacterium acnes infection.

In August 2025, the patient was admitted for periprosthetic infection after right hip arthroplasty in our hospital. On day seven of admission, vancomycin (1 g administered by intravenous infusion every 12 h) was started. On day 13, he underwent debridement. Over the 11 days of vancomycin therapy, hemoglobin decreased from 14.1 g/dL to 10.4 g/dL (red blood cell count from 4.56×10¹²/L to 3.47×10¹²/L), while platelet count increased from 213×10⁹/L to 385×10⁹/L. Indirect bilirubin rose from 3.2 μmol/L to 5.2 μmol/L, prothrombin time from 12.5 s to 14.0 s, D-dimer from 0.19 μg/mL to 2.45 μg/mL, and fibrinogen degradation products from 1.47 μg/mL to 6.92 μg/mL (Table 1). However, no definitive diagnosis of hemolysis was made at that time.

**Table 1:** Changes in laboratory parameters for first vancomycin exposure. RBC: red blood cell; HGB: hemoglobin; PLT: platelets; IBIL: indirect bilirubin; PT: prothrombin time; APTT: activated partial thromboplastin time; INR: international normalized ratio; FDP: fibrinogen degradation products

Lab parameters	Admission day	Eleven days after vancomycin treatment	Reference range
RBC (×10¹²/L)	4.56	3.47	4.3-5.8
HGB (g/dL)	14.1	10.4	13-17.5
PLT (×10⁹/L)	213	385	100-300
IBIL (μmol/L)	3.2	5.2	≤20.0
PT (s)	12.5	14.0	11-14
APTT (s)	28.7	30.9	27-45
INR	1.00	1.12	0.82-1.28
FDP (μg/mL)	1.47	6.92	0-5
D-dimer (μg/mL)	0.19	2.45	0-0.5

In February 2026, the patient was readmitted for right total hip arthroplasty revision, which was performed on day five. Joint fluid culture grew a non-fermenting bacillus sensitive to vancomycin. On day eight, vancomycin (1 g administered by intravenous infusion every 12 h) was reintroduced. Three days later, he developed sudden palpitations, chills, and occasional restlessness. Hemoglobin dropped sharply from 14.9 g/dL to 6.7 g/dL (red blood cell count from 4.82×10¹²/L to 2.19×10¹²/L), a decrease of 8.2 g/dL. Coagulation parameters showed prothrombin time 19.4 s, activated partial thromboplastin time 53.5 s, INR 1.57, and D-dimer 1.78 μg/mL (Table 2). Vancomycin was discontinued immediately. No other medical interventions were provided, as the patient remained hemodynamically stable and did not develop life-threatening anemia.

**Table 2:** Changes in laboratory parameters for secondary vancomycin exposure. RBC: red blood cell; HGB: hemoglobin; PLT: platelets; IBIL: indirect bilirubin; PT: prothrombin time; APTT: activated partial thromboplastin time; INR: international normalized ratio; FDP: fibrinogen degradation products

Lab parameters	Admission day	Three days after vancomycin treatment	Ten days after vancomycin withdrawal	Reference range
RBC (×10¹²/L)	4.82	2.19	3.49	4.3-5.8
HGB (g/dL)	14.9	6.7	10.8	13-17.5
PLT (×10⁹/L)	188	202	468	100-300
IBIL (μmol/L)	11.7	15.9	11.0	≤20.0
PT (s)	13.7	19.4	27.9	11-14
APTT (s)	29.2	53.5	42.6	27-45
INR	1.10	1.57	2.29	0.82-1.28
FDP (μg/mL)	4.14	7.13	12.74	0-5
D-dimer (μg/mL)	1.48	1.78	4.68	0-0.5

One day after vancomycin withdrawal, the direct antiglobulin test was positive, the reticulocyte percentage was 6.13%, and the absolute reticulocyte count was 0.155×10¹²/L (Table 3). Ten days after withdrawal, hemoglobin rebounded to 10.8 g/dL (red blood cell count 3.49×10¹²/L), and reticulocyte percentage rose to 7.00% (absolute count 0.244×10¹²/L). However, coagulation function worsened as follows: prothrombin time 27.9 s, international normalized ratio (INR) 2.29, activated partial thromboplastin time 42.6 s, D-dimer 4.68 μg/mL, and platelet count increased to 468×10⁹/L (Table 2). Rivaroxaban (5 mg once daily, which was initiated nine days after the operation) was discontinued. No further hemolysis occurred, and the patient’s hemoglobin gradually recovered without additional immunosuppressive therapy. A timeline of clinical events is summarized in Table 4.

**Table 3:** Key laboratory tests. DAT: direct antiglobulin test; RET%: reticulocyte percentage; RET#: absolute reticulocyte count

Lab parameters	Four days after vancomycin treatment	Ten days after vancomycin withdrawal	Reference range
DAT	Positive	-	-
RET%	6.13	7.00	0.5-1.5
RET# (×10^12^/L)	0.155	0.244	0.024-0.084

**Table 4:** Timeline of clinical events and vancomycin exposures. ASO: antistreptolysin O; RF: rheumatoid factor; INR: international normalized ratio; PLT: platelets; RET%: reticulocyte percentage; PT: prothrombin time

Time points	Event description	Hemoglobin (g/dL)	Key laboratory findings
August 2025	First admission for periprosthetic infection	14.1	ASO 105.03 IU/mL; RF 11.66 IU/mL
Day 7 of first admission	Vancomycin initiated	-	-
11 days after vancomycin	Hemoglobin decline noted	10.4	PLT 385×10⁹/L; D-dimer 2.45 μg/mL
September 2025	Vancomycin discontinued (no definite diagnosis)	-	-
February 2026	Second admission for revision arthroplasty	14.9	Pre-operative screening: HBV/HCV/HIV/syphilis negative
Day 8 of second admission	Vancomycin re-exposed	-	-
Three days after re-exposure	Palpitations, chills, acute hemoglobin drop	6.7	PT 19.4s
One day after withdrawal	DAT confirmed positive	-	RET% 6.13
10 days after withdrawal	Hemoglobin rebounded; coagulopathy worsened	10.8	RET% 7.00; PLT 468×10⁹/L; PT 27.9s; INR 2.29

## Discussion

Vancomycin‑induced immune hemolytic anemia is a rare but potentially life-threatening adverse drug reaction. As of 2026, only a few case reports of such instances have been documented in the literature. This study presents a systematic review of the major published cases, summarizing their clinical characteristics, diagnostic key points, and treatment strategies. A summary of previously reported cases of vancomycin‑induced immune hemolytic anemia is presented in Table 5.

**Table 5:** Summary of reported cases.

S. no.	Studies	Age	Sex	Indication	Exposure route	Time of onset	Nadir hemoglobin (g/dL)	Platelet	DAT results	Vancomycin- dependent antibodies	Treatment and outcomes
1	González et al. (2003) [9]	One case complicated by AIDS. Vancomycin-dependent antibody was detected. Detailed clinical information was not provided.									
2	Gniadek et al. (2018) [10]	61	F	Postoperative infection after knee arthroplasty	IV+ spacer（1g)	7	5.0	Normal	Positive	Detected	Hemolysis was relieved after discontinuation of vancomycin.
3	Sudhakaran et al. (2019) [11]	72	M	Postoperative infection after hip arthroplasty	IV+ spacer（6g)	14	5.7	Not provided	Not tested	Not tested	Discontinuation of intravenous vancomycin was ineffective. Hemoglobin stabilized after surgical removal of bone cement.
4	Siddiqui et al. (2023) [12]	75	M	Knee joint infection	IV	3	5.4	Normal	Positive	Not tested	Hemoglobin rebounded three days after discontinuation of vancomycin.
5	Song et al. (2025) [5]	58	M	Postoperative infection after knee arthroplasty	IV (previous exposure history)	6	4.2	Normal	Positive	Detected	Improvement was observed after discontinuation of vancomycin and use of glucocorticoids and immunoglobulin.
6	Peng and Gao (2025) [13]	64	F	Postoperative knee revision	IV	7	3.4	Normal	Positive	Not tested	Improvement was observed after discontinuation of vancomycin and use of methylprednisolone, immunoglobulins, plasma exchange, and washed red blood cell transfusions.
7	Xie et al. (2025) [14]	50	M	Postoperative infection after knee arthroplasty	IV+ spacer	8	4.4	Normal	Positive	Detected	Death from cerebral infarction occurred the day after discontinuation of vancomycin.

These reported cases demonstrate that vancomycin-induced immune hemolytic anemia exhibits certain characteristic patterns. Hemolysis typically occurs seven to 14 days after initial drug exposure [10,11,13], while the onset time for re-exposure is significantly shortened, with severe hemolysis potentially developing within three to six days [5,12]. In our case, the patient experienced a sharp drop in hemoglobin levels within three days after re-exposure, consistent with the features of an immune memory-driven "recall response." Hemoglobin levels decline acutely and rapidly, potentially decreasing by 5-6 g/dL within 24 h [13]. In severe cases, they may drop to 3.4-5.7 g/dL, necessitating emergency blood transfusion intervention [5,10-14]. In our case, hemoglobin levels decreased by 8.2 g/dL, confirming an extremely high risk of re-exposure. The platelet counts remained normal in all confirmed cases, which is a key distinguishing feature of DIIHA from conditions such as thrombotic thrombocytopenic purpura (TTP) and disseminated intravascular coagulation (DIC). All cases tested positive for DAT, typically showing IgG and/or C3 positivity [5,10,12-14]. Peng and Gao reported a significantly elevated reticulocyte count (13.15%), while in the present case, it was 7.00%, reflecting good bone marrow compensatory function [13]. Lactate dehydrogenase (LDH) levels were significantly elevated (Peng and Gao reported 3870 U/L [13]; Xie et al. reported 1080 U/L [14]). Indirect bilirubin levels were elevated (reported by Xie et al. as 131.4 μmol/L; in the present case, 15.9 μmol/L, indicating a mild increase) [14]. Using the Naranjo probability scale, our case scored seven points, suggesting a probable causal relationship between vancomycin re-exposure and immune hemolytic anemia. Sudhakaran et al. first reported a case of persistent hemolysis caused by vancomycin-containing bone cement [11]. The patient continued to experience hemolysis after discontinuation of intravenous vancomycin until hemoglobin levels stabilized following surgical removal of the vancomycin-containing bone cement [11]. This suggests that localized high concentrations of vancomycin can also persistently stimulate antibody production, leading to refractory hemolysis.

DIIHA is primarily mediated through the "drug-dependent antibody" mechanism. As a hapten, vancomycin non-covalently binds to the erythrocyte membrane to form neoantigens, stimulating the host to produce specific antibodies (mainly IgG). Upon re-exposure to vancomycin, the antibodies bind to drug-erythrocyte complexes, activating the complement system and leading to intravascular and extravascular hemolysis [15]. Gniadek et al. demonstrated that vancomycin-dependent antibodies only react with erythrocytes in the presence of soluble drugs, but not with erythrocytes pre-treated with vancomycin [10]. This indicates that vancomycin binds to erythrocyte membranes through non-covalent interactions, making it readily removable during washing processes [10]. Xie et al. first discovered that vancomycin-dependent antibodies exhibit specificity for blood group N antigens, with antibody titers showing a positive correlation with N antigen expression intensity, providing novel insights into the fine specificity of drug antibodies [14].

Most patients have a favorable prognosis, with hemoglobin levels typically beginning to recover within one to two weeks after timely discontinuation of medication. The case reported by Xie et al. resulted in death from cerebral infarction the day after drug withdrawal, suggesting that severe hemolysis can induce a hypercoagulable state and increase the risk of thrombotic events [14].

In the present case, after discontinuation of vancomycin, the patient's coagulation function (PT: 27.9s, INR: 2.29) deteriorated, with D-dimer levels rising to 4.68 μg/mL and platelet count increasing to 468×10⁹/L. This may be a result of a combination of multiple factors, such as persistent activation of the coagulation system due to non-fermenting bacillus infection [16]. Hypercoagulability following hemolysis, and severe hemolysis releasing erythrocyte contents, which activated the coagulation cascade [17]. Postoperative use of rivaroxaban in the patient significantly prolonged the PT/INR ratio [18]. Reactive thrombocytosis, as a compensatory response to inflammation and hemolysis, inherently increases thrombotic risk. The case reported by Xie et al., who died of cerebral infarction, serves as a warning that hypercoagulability may occur following severe hemolysis, necessitating active investigation for thrombotic events [14].

## Conclusions

This case shows that vancomycin can induce immune hemolytic anemia, which may become more severe and rapid upon re-exposure. Clinicians should consider drug-induced immune hemolytic anemia in patients presenting with acute, unexplained anemia and normal platelet counts. A positive DAT is a key diagnostic finding and should be obtained promptly. Once vancomycin-associated hemolysis is confirmed, permanent discontinuation of the drug should be strongly considered, and the reaction clearly documented in the patient’s medical record.
